# Solving the inverse problem in electrocardiography imaging for atrial fibrillation using various time-frequency decomposition techniques based on empirical mode decomposition: A comparative study

**DOI:** 10.3389/fphys.2022.999900

**Published:** 2022-11-02

**Authors:** Zhang Yadan, Lian Xin, Wu Jian

**Affiliations:** Research Center of Biomedical Engineering, Institute of Biopharmaceutical and Health Engineering, Tsinghua Shenzhen International Graduate School, Shenzhen, China

**Keywords:** electrocardiography imaging, inverse problem, EMD-based solutions, time-frequency decomposition, atrial fibrillation

## Abstract

Electrocardiographic imaging (ECGI) can aid in identifying the driving sources that cause and sustain atrial fibrillation (AF). Traditional regularization strategies for addressing the ECGI inverse problem are not currently concerned about the multi-scale analysis of the inverse problem, and these techniques are not clinically reliable. We have previously investigated the solution based on uniform phase mode decomposition (UPEMD-based) to the ECGI inverse problem. Numerous other methods for the time-frequency analysis derived from empirical mode decomposition (EMD-based) have not been applied to the inverse problem in ECGI. By applying many EMD-based solutions to the ECGI inverse problem and evaluating the performance of these solutions, we hope to find a more efficient EMD-based solution to the ECGI inverse problem. In this study, five AF simulation datasets and two real datasets from AF patients derived from a clinical ablation procedure are employed to evaluate the operating efficiency of several EMD-based solutions. The Pearson’s correlation coefficient (CC), the relative difference measurement star (RDMS) of the computed epicardial dominant frequency (DF) map and driver probability (DP) map, and the distance (Dis) between the estimated and referenced most probable driving sources are used to evaluate the application of various EMD-based solutions in ECGI. The results show that for DF maps on all simulation datasets, the CC of UPEMD-based and improved UPEMD (IUPEMD)-based techniques are both greater than 0.95 and the CC of the empirical wavelet transform (EWT)-based solution is greater than 0.889, and the RDMS of UPEMD-based and IUPEMD-based approaches is less than 0.3 overall and the RDMS of EWT-based method is less than 0.48, performing better than other EMD-based solutions; for DP maps, the CC of UPEMD-based and IUPEMD-based techniques are close to 0.5, the CC of EWT-based is 0.449, and the CC of the remaining EMD-based techniques on the SAF and CAF is all below 0.1; the RDMS of UPEMD-based and IUPEMD-based are 0.06∼0.9 less than that of other EMD-based methods for all the simulation datasets overall. On two authentic AF datasets, the Dis between the first 10 real and estimated maximum DF positions of UPEMD-based and EWT-based methods are 212∼1440 less than that of others, demonstrating these two EMD-based solutions are superior and are suggested for clinical application in solving the ECGI inverse problem. On all datasets, EWT-based algorithms deconstruct the signal in the shortest time (no more than 0.12s), followed by UPEMD-based solutions (less than 0.81s), showing that these two schemes are more efficient than others.

## 1 Introduction

The recurrence of atrial fibrillation (AF) after ablation, particularly persistent AF, remains a great challenge (Hussein, 2020). The location of the drivers that triggers and maintains AF has not been accurately identified (Pellman and Sheikh, 2015). As a result, improving the efficiency and accuracy of source mapping of AF driving sources is a pressing issue that must be addressed (Dubois et al., 2015).

Electrocardiographic imaging (ECGI) can non-invasively reconstruct epicardial potential from body surface electrocardiographic signals (ECGs) based on geometric structural data of the heart and torso (Cluitmans et al., 2018). By further calculating the isochronous map, domain frequency (DF) map, and driver probability (DP) map on the atria surface, the location of AF drivers can be more intuitively presented (Dubois et al., 2015; Figuera et al., 2016; Salinet et al., 2021). However, due to the limited number of body surface signals, there is an ill-posed problem in solving the ECGI inverse problem (Cluitmans et al., 2015).

Numerous regularization solutions have been proposed to solve this ill-posed problem at present. A pioneering solution for the ill-posed problem is the Tikhonov regularization, which is strongly developed as a motivation for the regularization theory (Wang, 2012; Benning and Burger, 2018). Besides, the truncated singular value decomposition (TSVD) and its modified version based on a singular value decomposition process are gradually popular (Caulier-Cisterna et al., 2018). However, these techniques have not yet achieved reliable and stable epicardial potential reconstructions. These techniques do not screen the different components of the ECGs from the perspective of multiscale time-frequency decomposition, which could skew the reconstruction of the epicardial potential. In our previous study, we performed multi-scale decomposition of body surface signals using the Uniform Phase Mode Decomposition (UPEMD) technique (Zhang et al., 2022), which was derived from Empirical Mode Decomposition (EMD) (Wang et al., 2018). Then, based on the various time-frequency domain features of the decomposed signal, alternative regularization techniques are implemented for the portions comprising various ECG information. Different weights are then assigned to the regularization findings of each component, which substantially increases the accuracy and robustness of the inverse issue solution. Please reference (Zhang et al., 2022) for a more thorough explanation of this technique.

Although we have studied the solution to the inverse problem based on UPEMD in ECGI, various other methods for multi-scale decomposition of signals developed based on EMD have not yet been applied to the inverse problem solving, which is worthy of further exploration.

EMD can decompose ECGs into several parts in the time-frequency domain, but there will be mode mixing and residual noise. In order to solve the above problems, many improved algorithms based on EMD (EMD-based) have been proposed. Among them, in recent years, multivariate empirical mode decomposition (MEMD) (Zheng and Xu, 2019), noise-assisted MEMD (NAMEMD) (Ahrabian et al., 2012), variational mode decomposition (VMD) (Dragomiretskiy and Zosso, 2014), successive variational mode decomposition (SVMD) (Nazari and Sakhaei, 2020), empirical wavelet transform (EWT) (Gilles, 2013; Hurat, 2020), UPEMD (Wang et al., 2018), improved UPEMD (IUPEMD) (Hurat, 2020; Ying et al., 2021; Zheng et al., 2021) have been proposed successively. The effectiveness of the above methods has been verified in mechanical fault detection, voice signal, ECG signal, and seismic signal processing (Lal et al., 2018; Zeng and Yuan, 2021; Liu et al., 2022). In order to study the influence of different EMD-based solutions on the accuracy and reliability of ECGI inverse operation, firstly, the ECGs were decomposed using the various EMD-based solutions mentioned above in this paper. Secondly, the truly useful components for the inverse operation were then screened out using the same principle. Finally, the same post-processing was carried out on the various parts screened out using the various EMD-based solutions to obtain the final epicardial potential.

The remaining of the study is organized as follows. Section 2 describes the principle of the inverse problem, the algorithm principles of various EMD-based technologies, the research process of this paper, and the solutions to the inverse problem based on EMD-based solutions. Section 3 focuses on introducing the data source used in the experiment, the parameter selection of the algorithm, the evaluation index of the algorithm, and the experimental results of various EMD-based technologies. Section 4 is a discussion of the results. The conclusion is presented in section 5.

## 2 Methods

### 2.1 Inverse problem

ECGI can non-invasively reconstruct the electrical signal on the heart surface based on the high-density ECGs (Borras and Chamorro-Servent, 2021; Salinet et al., 2021). The mathematical mechanism of the ECGI inverse problem can be expressed as follows.

$$\Phi_{H}=A^{-1}*\Phi_{B}$$ (1)

Where **A** is the transfer matrix, indicating the conduction of electrical signals from the epicardium to the body surface, and $\Phi_{B}$ is the ECGs, $\Phi_{H}$ is the epicardial potential (Potyagaylo et al., 2021). The transfer matrix is determined by geometric models of the atrium and torso in this study (Zhou et al., 2018).

Even though the measurement error in the inverse problem’s solution for $\Phi_{B}$ is minimal, the inverse problem’s ill-posedness results in a substantial calculation deviation on $\Phi_{H}$ . The Tikhonov regularization is a typical remedy for this issue, which can be mathematically represented as follows (Tikhonov, 1963).

$$\Phi_{H}=argmin\left\{{\left\|\Phi_{B}-A\Phi_{H}\right\|}_{2}+{\lambda \left\|\Lambda \Phi_{H}\right\|}_{2}\right\}$$ (2)

Among them, **Λ** is the regularization operator, and *λ* is the regularization parameter, which is typically determined by the L-curve method (Orozco Rodríguez, 2011; Aster et al., 2018). (Chen et al., 2019) presents a novel L-curve technique based on bilateral accumulative area detector (L-BAA). BAA is a curve feature point identification operator that offers superior anti-interference capabilities. Tikhonov regularization can improve the smoothness and stability of the inverse operation. TSVD regularization, on the other hand, has a greater ability to suppress noise. The mathematical principle underlying TSVD is given as follows (Wu et al., 2013).

$$\Phi_{H}=argmin\left\|A_{k}\Phi_{H}-\Phi_{B}\right\|$$ (3)

*k* is the truncation parameter, and like Tikhonov, it may be found using the L-BAA method. In addition to the influence of the regularization method on the result of the inverse operation, $\Phi_{B}$ has a direct effect on $\Phi_{H}$ .

### 2.2 EMD-based signal decomposition solutions

To improve the accuracy of $\Phi_{H}$ , EMD-based technology can sieve the different time-frequency components of the ECGs $\Phi_{B}$ , assist in extracting specific components from $\Phi_{B}$ to participate in the inverse operation and suppress the other part that has a little positive effect on the inverse operation. Currently, developed EMD-based technologies primarily include the following.

#### 2.2.1 Variational mode decomposition

VMD decomposes the raw signal into narrowband signals with separate bands squeezed around different center frequencies in a non-recursive manner (Dragomiretskiy and Zosso, 2014; Zosso et al., 2017). In VMD, the setting of the optimal number of modes *k*, and weight factor α are two key parameters. The larger *k*, the heavier the computational burden the method has. The smaller the *k*, the more severe the mode aliasing may be. The smaller α, the larger the bandwidth of each component. VMD defines intrinsic mode function (IMF) as a limited bandwidth amplitude-modulation-frequency-modulation (AM-FM) signal, requiring that the sum of each IMF’s estimated bandwidths is the smallest, and that the sum of all IMFs is equal to the original signal (Isham et al., 2019). For more detailed mathematical principles and implementation steps of the algorithm (Dragomiretskiy and Zosso, 2014), please refer to the appendix.

#### 2.2.2 Successive variational mode decomposition

(Nazari and Sakhaei, 2020) presented SVMD to discover the optimal k with the use of a heuristic method to adaptively select the best number of modes *k* and the weighting factor in VMD. In contrast to VMD, SVMD incorporates a new penalty function to lessen spectral overlap (Guo et al., 2022).

VMD extracts modes concurrently whereas SVMD extracts all IMFs sequentially. SVMD has lower computational complexity than VMD. SVMD operates by constantly applying VMD to the signal until the decomposition error reaches a certain threshold; this succession aids in accelerating convergence and avoiding the extraction of undesired modes. In SVMD, just an initial and maximum weighting factor is required to be established in advance (Wang and Zhou, 2021). Please see the appendix for further information on the algorithm’s implementation steps and more precise mathematical foundations.

#### 2.2.3 Multivariate empirical mode decomposition

When employing an EMD-based technique for multivariate signal decomposition, the amount of IMFs decomposed from various channel signals may vary, impeding the subsequent synchronization analysis of decomposed multichannel signals. MEMD was suggested by (Rehman and Mandic, 2010) as a solution to the issue based on EMD. Using a low discrepancy Hammersley sequence, the raw multivariate signal is first projected into n-dimensional space in MEMD, and the projection signal is then decomposed by EMD to generate the same number of IMFs for multichannel signals. (Rehman and Mandic, 2010; Bussett, 2021). The appendix has more information about the algorithm and how to put them into practice.

#### 2.2.4 Noise-assisted multivariate empirical mode decomposition

Rehman and Mandic (Rehman et al., 2013) also proposed that NAMEMD can also decompose multi-channel signals into the same number of modes. Compared with MEMD, NAMEMD adds independent white noise to the signal to be decomposed to improve the problems of modal aliasing and residual noise (Zhao et al., 2022). Appendix contains the algorithm implementation procedures.

#### 2.2.5 Uniform phase empirical mode decomposition

In order to get rid of the modal splitting and margin noise present in noise-assisted EMD, UPEMD adds a sinusoidal signal with a uniform phase (Wang et al., 2018). There will be some empirical mistakes introduced since the sinusoidal signal added to UPEMD’s magnitude and phase are governed by subjective experience. See appendix for algorithm implementation details.

#### 2.2.6 Improved uniform phase empirical mode decomposition

To acquire the added sinusoidal signal’s ideal amplitude and to increase the decomposition’s accuracy, IUPEMD applies an orthogonality index to choose the optimal amplitude from numerous decomposition results under various amplitudes. Moreover, the mean curve is not able to be completely isolated from the signal unless the iterative residual signal has been updated, while IUPEMD employs minimum orthogonality as an ideal weight selection criterion to make the mean curve separated from the signal to reduce the residual (Ying et al., 2021; Zheng et al., 2021). Appendix contains algorithm implementation steps.

#### 2.2.7 Empirical wavelet transform

The wavelet transform approach has a positive impact on multi-resolution signal analysis. By merging EMD and wavelet analysis theory, some researchers have developed EWT. EWT generates an adaptive wavelet to extract AM and FM components. First, previous knowledge is extracted from the original signal’s Fourier spectrum, and the signal spectrum is adaptively subdivided based on the distribution of extreme points in the frequency domain; next, a wavelet filter bank is formed. Finally, the empirical wavelet transform, i.e. band-pass filtering, is implemented in the divided spectrum range in order to separate individual FM and AM components, and the spectrum of these components is supported firmly (Gilles, 2013; Kedadouche et al., 2016; Liu et al., 2016). Please refer to the appendix for a more comprehensive description of the algorithm’s implementation procedure.

The comparative study on EMD-based solutions to the inverse problem.

In this comparative study, various EMD-based solutions are first employed to accomplish multi-scale decomposition on N ECG signals in the EMD-based solution of the inverse issue. The N decomposed signal groups are represented as $S_{1}$ , $S_{2}$ ,…, $S_{n}$ , and each **S** comprises *m* decomposed signals.

Secondly, the decomposed signal will be screened. The *m* signals are separated into two parts, the valuable part $P_{1}$ and the less valuable signals $P_{2}$ , where the decomposed signals with a higher variance contribution rate and the correlation coefficient are treated as valuable parts and the rest of the decomposed signals are taken as the less valuable signals. Then, the $P_{1}$ parts of the N decomposed signals are then combined to form $\Phi_{B1}$ , and the $P_{2}$ parts are combined to form $\Phi_{B2}$ .

Thirdly, utilizing Tikhonov and TSVD regularization for the two parts $\Phi_{B1}$ and $\Phi_{B2}$ to produce the inverse solutions $\Phi_{H1}$ and $\Phi_{H2}$ .

$$\Phi_{H1}=argmin\left\{{\left\|\Phi_{B1}-A\Phi_{H1}\right\|}_{2}+{\lambda \left\|\Lambda \Phi_{H1}\right\|}_{2}\right\}$$ (4)

$$\Phi_{H2}=argmin\left\|A_{k}\Phi_{H2}-\Phi_{B2}\right\|$$ (5)

$$\Phi_{H}=\alpha *\Phi_{H1}+\left(1-\alpha \right)*\;\Phi_{H2}$$ (6)

**FIGURE 1:**
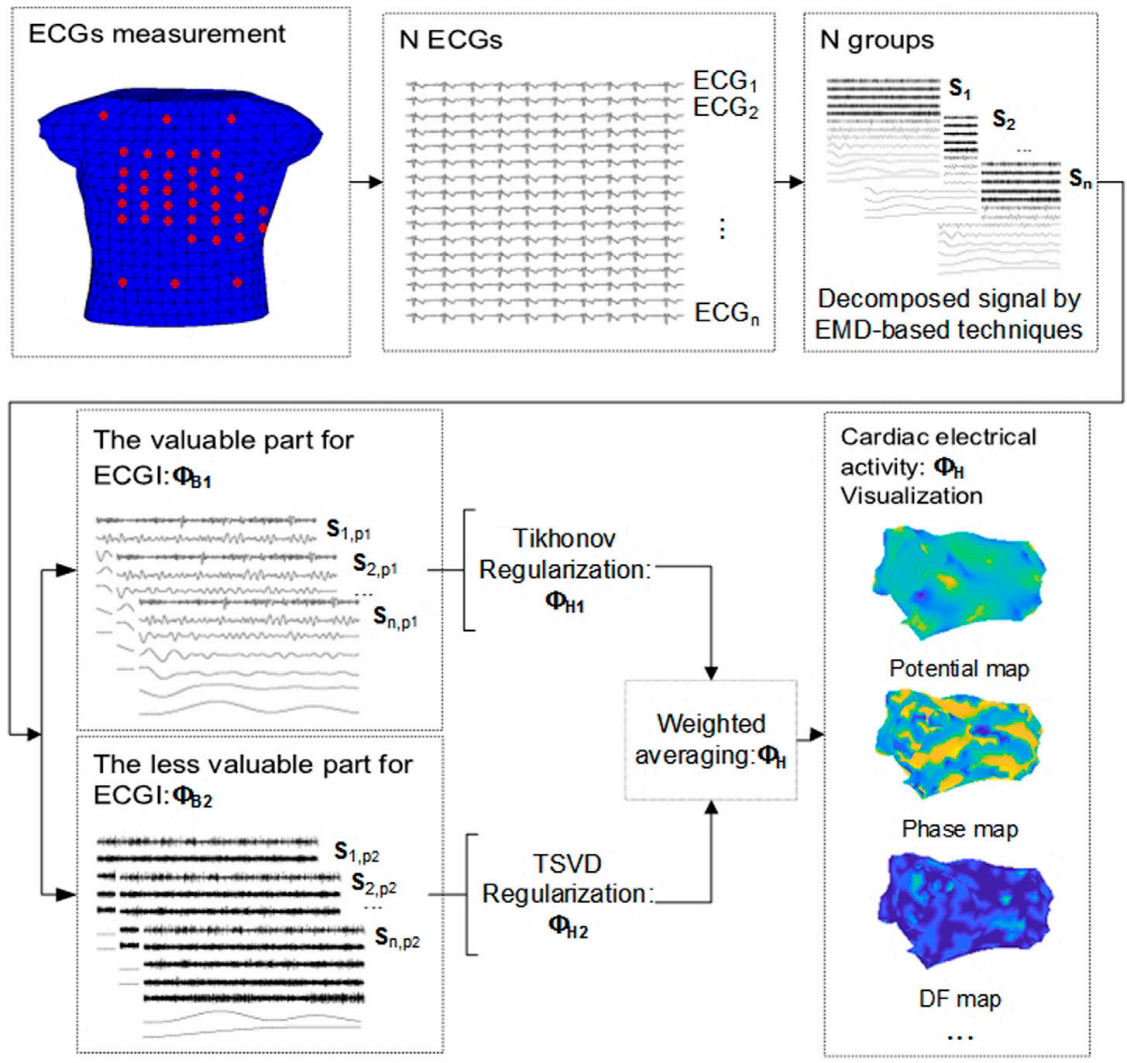
EMD-based solution to the inverse problem in ECGI.

Finally, weighting and averaging $\Phi_{H1}$ and $\Phi_{H2}$ to obtain the potential signal on the epicardium, $\Phi_{H}$ . The literature (Yadan et al., 2022) contains more detailed description about these steps. The entire procedure is depicted in Figure 1.

The EMD-based technologies in ECGI are evaluated based on the computational efficiency and precision of inverse solutions.

## 3 Results

### 3.1 Datasets

To compare and test several proposed EMD-based solutions for the inverse problem in ECGI, two simulation datasets containing a total of five different AF types and two datasets from real AF patients were used in this study. One of the simulated datasets is from (Figuera et al., 2016; Pedron-Torrecilla et al., 2016), including two different AF propagation patterns, simple AF (SAF) and complex AF (CAF) on the atrial surface. The other simulated dataset from the EDGAR project^1^ (Aras et al., 2015) contains realistic mathematical models of the two atria and the torso with different AF impulse propagation patterns. AF was simulated by a driving rotor on either the left atrium (LA) or right atrium (RA) and fibrillatory conduction to the rest of the atria, and in the case of LA driver, with and without fibrotic conduction (LA_normal and LA_fibrotic); The dataset is called RA_normal where the driver is located in RA without fibrotic conduction. Torso potentials were computed by solving the forward problem of electrocardiography (Pedron-Torrecilla et al., 2016).

The real AF dataset from the EDGAR project consists of the signals and geometrical meshes from two AF patients (Patient one# and Patient 2#) derived for an ablation procedure. ECGs were recorded simultaneously with the endocardial recordings with high-resolution multipolar catheters. This genuine AF dataset consists of 62-channel (Patient 1#) and 72-channel (Patient 2#) intracardiac catheter mapping signals from two AF patients, 54-lead body surface ECGs, atrial torso geometry, and transmission matrix A.

### 3.2 Parameters selection

We determined the initial values of various parameters by comprehensive tests, together with the sampling frequency and features of ECGs. The ECGs are decomposed into 10 layers respectively in VMD, i.e. k=10 . In SVMD, the number of decomposition layers of ECGs totally depends on the different datasets, ranging from 5 to 19. All ECGs in MEMD are adaptively decomposed into 11 layers. In NAMEMD, when the added white noise standard deviation is 1, the decomposition layers of ECGs are shown in Table 1 for different datasets. Furthermore, the ECGs are split into 10 layers using EWT. The amplitude of the sinusoidal signal added in UPEMD is set to one for all datasets, and the optimal number of phases is 18. In general, IUPEMD determines the number of phases between 4 and 32, and the optimal amplitude of the sinusoidal signal is set between 0.15 and 0.4. Table 2 shows the optimal signal amplitude and phase quantity selected adaptively by IUPEMD for various datasets.

**TABLE 1:** The decomposition layers of ECGs for different datasets in NAMEMD.

Dataset	SAF	CAF	LA_fibrotic	LA_normal	RA_normal	Patient #1	Patient #2
Decomposition layers	12	11	11	12	11	13	14

**TABLE 2:** The optimal signal amplitude and phase quantity selected by IUPEMD for different datasets.

Dataset	SAF	CAF	LA_fibrotic	LA_normal	RA_normal	Patient #1	Patient #2
Amplitude	0.17	0.17	0.15	0.15	0.2	0.15	0.25
Phase Quantity	4	4	8	8	4	4	4

After the ECGs have been decomposed using the aforementioned time-frequency decomposition solutions, the decomposed signals are divided into two parts based on the variance contribution rate and the correlation coefficient: one containing a large amount of irrelevant information and the other containing more useful information. In all of the inverse problem solutions shown in this work, the processing that follows is identical.

### 3.3 Evaluation index

In this study, Pearson’s correlation coefficient CC (Puth et al., 2014; Liu et al., 2021; Cui et al., 2022)and relative difference measurement star (RDMS) (Figuera et al., 2016) are used to quantify the similarity between the calculated and actual DF and the DP maps, thereby evaluating several EMD-based solutions to the inverse problem.

$$CC=\frac{\sum_{i=1}^{m}\left(x_{i}-\bar{x}\right)\left(y_{i}-\bar{y}\right)}{\sqrt{\sum_{i=1}^{m}{\left(x_{i}-\bar{x}\right)}^{2}}\sqrt{\sum_{i=1}^{m}{\left(y_{i}-\bar{y}\right)}^{2}}}$$ (4a)

$$RDMS=\sqrt{\underset{j}{\sum }{\left(\frac{x_{k}}{\left\|x^{2}\right\|}-\frac{\hat{x_{k}}}{\left\|{\hat{x}}^{2}\right\|}\right)}^{2}}$$ (5a)

In addition, to quantify the accuracy of the calculated DF or DP from many perspectives, the Euclidean distance (denoted by Dis) has also been employed (Etal, 2021). The calculated and referenced numbered positions of the n greatest DF or DP points will be processed as an n-dimensional vector, respectively. Dis measures the distance between these two vectors, that is, the similarity between the n biggest estimated DF or DP sites and their references.

$$Dis=\sqrt{\sum_{i=1}^{n}{\left(x_{i}-y_{i}\right)}^{2}}$$ (6a)

*n* denotes the number of sites. $x_{i}$ and $y_{i}$ are the calculated and referenced numbered positions of the n greatest DF or DP points, respectively. If n=10 , the Dis between the estimated and reference first 10 greatest DP location or the DF position will be calculated, representing the index distance between the estimated and the reference driving sources.

### 3.4 Experimental results

The DF and DP maps on the epicardium are constructed, and potential AF drivers are identified using several EMD-based solutions in this study.

#### 3.4.1 Results on the simulation datasets

**FIGURE 2:**
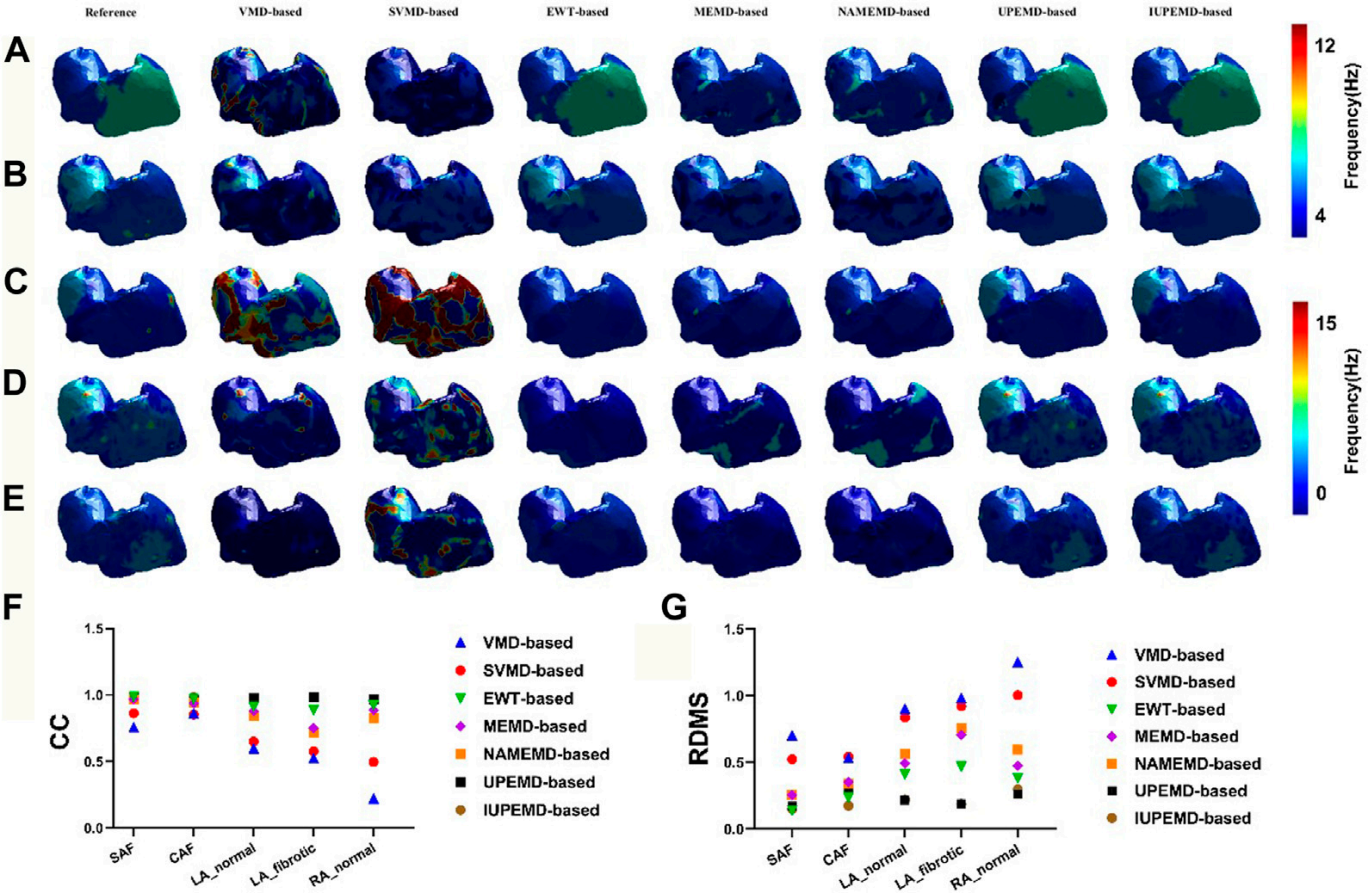
The DF maps using various EMD-based methods under the datasets SAF, CAF, LA_normal, LA_fibrotic, and RA_normal are represented by **(A–E)**, respectively. For each separate dataset, the reference DF map is in the first column, followed by the DF maps for the VMD-based, SVMD-based, EWT-based, MEMD-based, NAMEMD-based, UPEMD-based, and IUPEMD-based solutions, starting with the second column. On each model, a distinct color denotes the DF at that place. The RDMS **(G)** and CC **(F)** between the computed EMD-based algorithm and the real DF map for each EMD-based solution are shown as well.

Figure 2 shows the DF maps of seven different EMD-based solutions for five simulation datasets. Among them, the DF maps generated by the UPEMD-based and IUPEMD-based solutions are the most comparable to the real maps in all datasets; the DF under the EWT-based solution also exhibits a good resemblance to the real one for SAF and CAF. From the perspective of CC, for the UPEMD-based, IUPEMD-based, and EWT-based methods, the CC is higher than other methods. The CC of UPEMD-based and IUPEMD-based techniques are both greater than 0.95 for five distinct simulation datasets, while the CC of the EWT-based solution is greater than 0.915 for all simulation datasets excluding the LA_fibrotic dataset (CC on the LA_fibrotic: 0.889). The aforementioned three solutions have distinct advantages over other EMD-based technologies.

On these five simulation datasets, the RDMS of UPEMD-based and IUPEMD-based approaches is less than 0.3 overall. On the datasets SAF, CAF, and RA normal, the RDMS of EWT-based method is less than 0.4; however, on the datasets LA_normal and LA_fibrotic, the RDMS is 0.413 and 0.471, respectively. Both the RDMS of VMD-based and SVMD-based are more than 0.5. There is little difference between the DF maps for MEMD-based and NAMEMD-based solutions, whereas CC and RDMS for MEMD-based solutions are slightly better than those for NAMEMD-based solutions, indicating that when MEMD is used to decompose ECGs, the addition of white noise cannot improve the inverse results; the SVMD-based inverse solution is superior to VMD overall.

**FIGURE 3:**
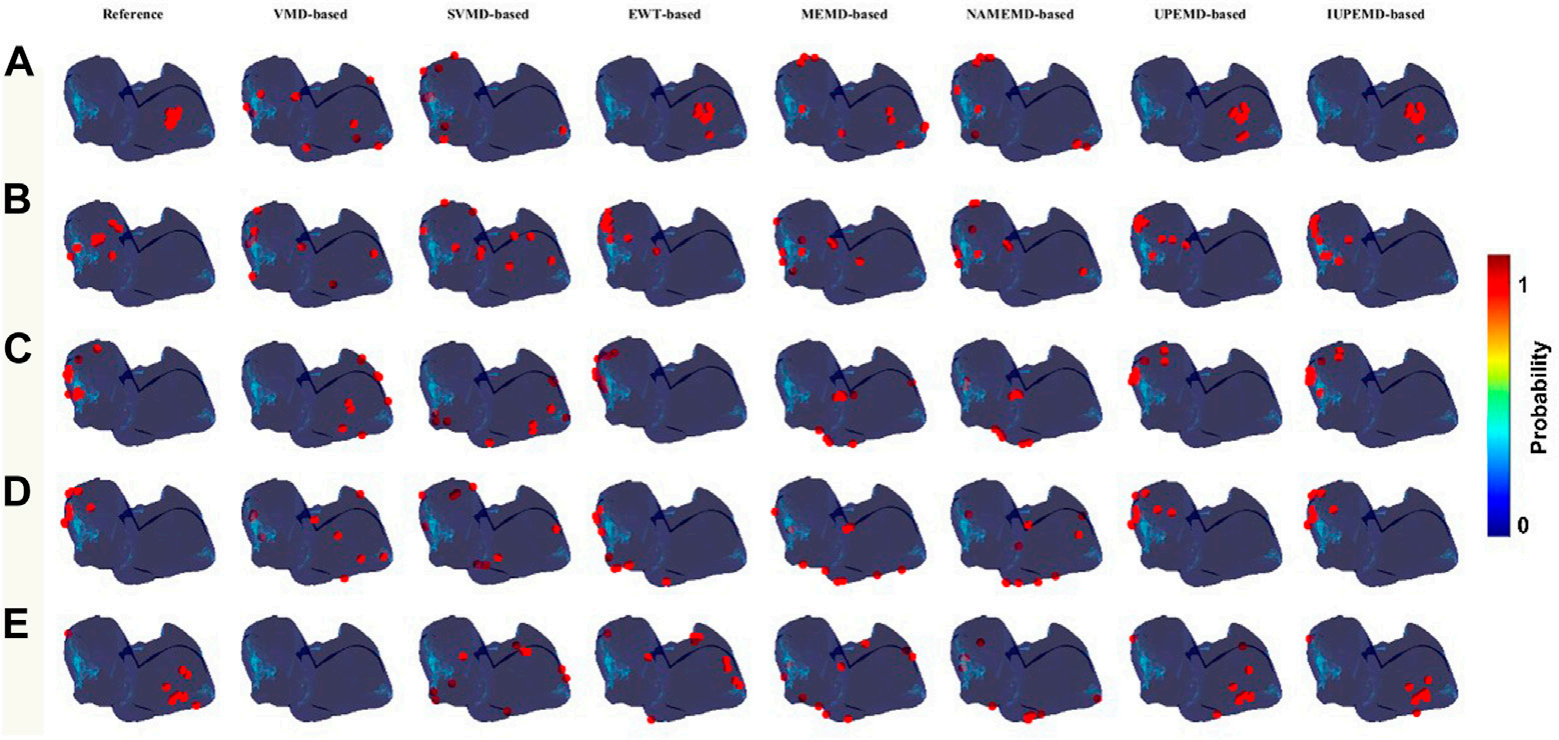
DP maps (red circles mark the 10 locations with the highest probability of driver location). Among them **(A–E)** represent the DP maps generated by various EMD-based algorithms for SAF, CAF, LA_normal, LA_fibrotic, and RA_normal, which from left to right correspond to the DP maps for VMD-based, SVMD-based, EWT-based, MEMD-based, NAMEMD-based, UPEMD-based, and IUPEMD-based solutions.

This paper calculates the DP maps for each algorithm and identifies ten drivers with the highest probability as well. The relevant details are shown in Figure 3. In general, DP maps of UPEMD-based and IUPEMD-based solutions are the most accurate, followed by EWT and other algorithms that have varying degrees of false detection. Compared to VMD-based and MEMD-based solutions, the inverse operation of the upgraded algorithms, SVMD-based and MEMD-based solutions, are not significantly enhanced. In the VMD-based scheme for the RA _normal, the VMD-based solution does not detect the driver, hence no driver position is marked in the VMD-based inverse solution in (E).

**FIGURE 4:**
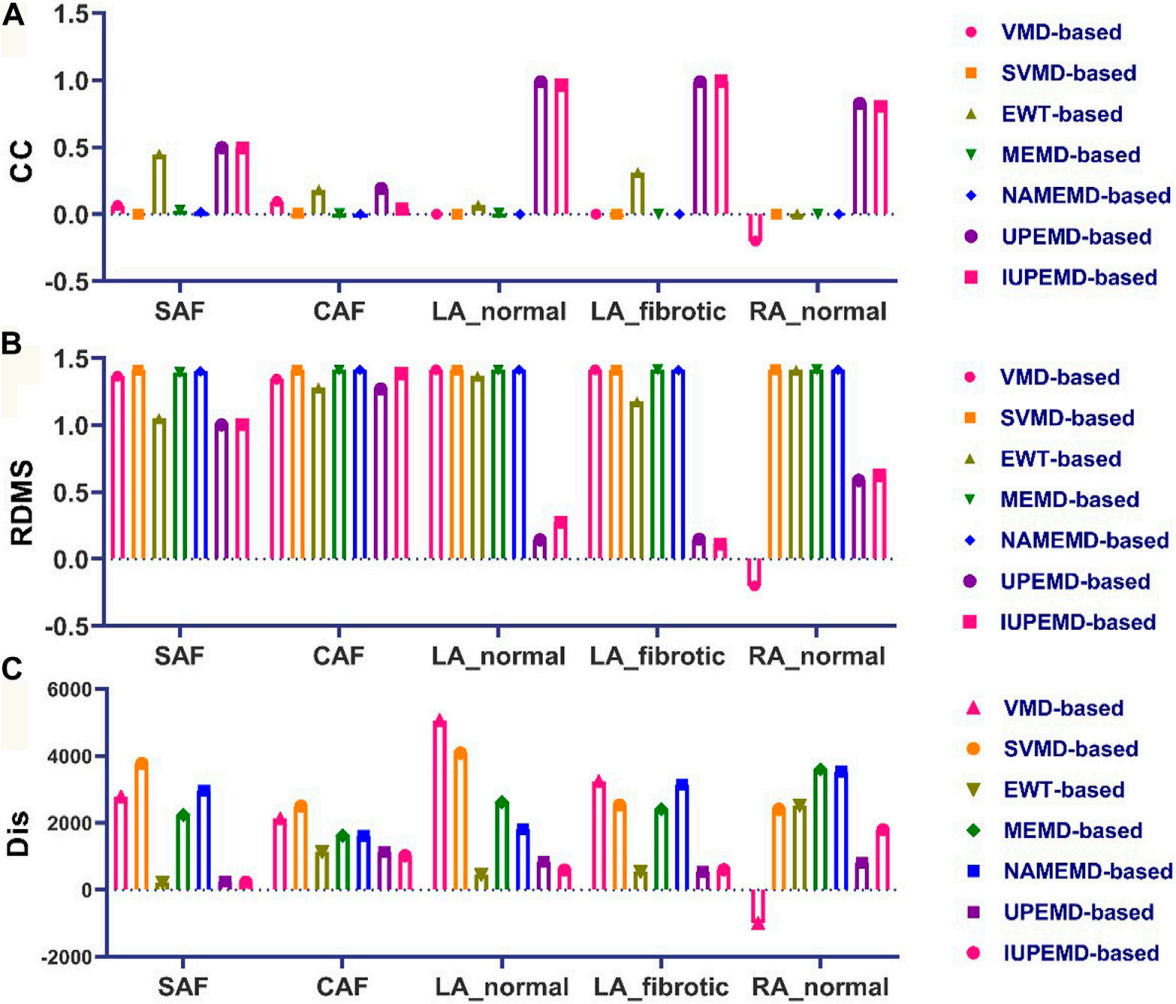
Quantitative evaluation of the DP map: **(A)** CC **(B)** RDMS **(C)** Dis (top 10 greatest possible drivers). The horizontal axis displays, from left to right, the quantitative indications of VMD-based, SVMD-based, EWT-based, MEMD-based, NAMEMD-based, UPEMD-based, and IUPEMD-based solutions for every dataset.

Meanwhile, Figure 4 depicts the CC, RDMS, and the Dis between the calculated drivers and the reference drivers on the DP maps. If no driving source is discovered, its CC, RDMS, and Dis are manually set to negative values. For DP maps, the CC of UPEMD-based (CC = 0.498) and IUPEMD-based (0.496) techniques are close to 0.5, the CC of EWT-based is 0.449, and the CC of the remaining EMD-based techniques on the SAF and CAF is all below 0.1; the RDMS of UPEMD-based and IUPEMD-based solutions are lower 0.3 on LA_normal and LA_fibrotic, while other EMD-based methods’ RDMS is more than 1.2. On the SAF and CAF datasets, the RDMS of UPEMD-based, IUPEMD-based, and EWT-based methods is likewise less than that of other EMD-based methods. These results show that the UPEMD-based, EWT-based and IUPEMD-based solutions outperform other algorithms in the CC and RDMS of DP.

As for the Dis between estimated and reference driving sources, in general, the drivers calculated by EWT-based, UPEMD-based, and IUPEMD-based solutions are the closest to the reference drivers; For all the datasets, the Dis for VMD-based and SVMD-based solutions show no noticeable advantages or disadvantages, as do MEMD-based and NAMEND-based solutions, indicating that these algorithms have limited efficacy in solving ECGI inverse problems.

#### 3.4.2 Results on the real datasets

**FIGURE 5:**
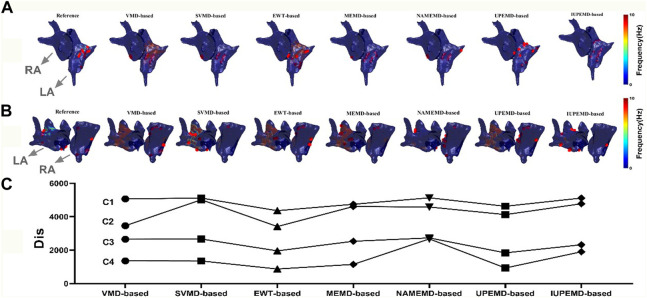
DF maps of two real patients with AF (red dots mark the 10 locations with the highest DF; both patients’ LA and RA are indicated by grey arrows). **(A)** DF maps for Patient 1#; **(B)** DF maps for Patient 2#; **(C)** The Dis between the computed and actual DF maps: C1, for Patient 1#, the Dis between the estimated and actual 62 maximum DF positions for various EMD-based solutions; C2, for Patient 2#, the Dis between the estimated and actual 73 maximum DF positions; C3, for Patient 1#, the Dis between the first 10 real and estimated maximum DF positions; C4, for Patient 2#, the Dis between the first 10 real and inversely calculated maximum DF positions.

This paper analyzes the DF maps of the epicardial potential generated by various EMD-based solutions on two authentic AF datasets and identified the 10 sites with the maximum DF, as shown in Figure 5 ((A) for patient one# and (B) for patient 2#). Figure 5A demonstrates that UPEMD-based and EWT-based solutions identified multiple maximum DF sites in the left atrium of patient 1#. Although these points were slightly dispersed in regard to the 10 maximum DF spots on the reference left atrium, the recognition results of UPEMD-based and EWT-based algorithms are more accurate than those of other EMD-based algorithms. For patient 2# (Figure 5B), both UPEMD-based and EWT-based algorithms recognized multiple highest DF spots in the RA. The maximum DF positions discovered by SVMD-based and IUPEMD-based approaches are mostly situated in the LA, which is much more than that of the reference LA, and they are definitely absent in the RA.

To analyze the aforementioned DF maps’ findings quantitatively, the Dis between the actual and measured maximum DF was determined (62 locations for patient one# and 73 sites for patient 2#). Also given is the disparity between the projected and actual top 10 maximum DF positions. In general, the Dis between the first 10 real and estimated maximum DF positions of UPEMD-based and EWT-based methods are less than 1960 for patient 1#, while that of other EMD-based techniques are all more than 2300; for patient 2#, the Dis between the first 10 real and inversely calculated maximum DF positions of UPEMD-based (Dis = 934) and EWT-based (Dis = 875) techniques are lower to 935, less than that of others (more than 1147). The UPEMD-based and EWT-based are superior to others even for the Dis between the actual and measured maximum DF (62 locations for patient one# and 73 sites for patient 2#), showing that the estimated epicardial abnormal area by the UPEMD-based and EWT-based solutions is closer to the reference one.

#### 3.4.3 The execution efficiency of EMD-based solutions

**FIGURE 6:**
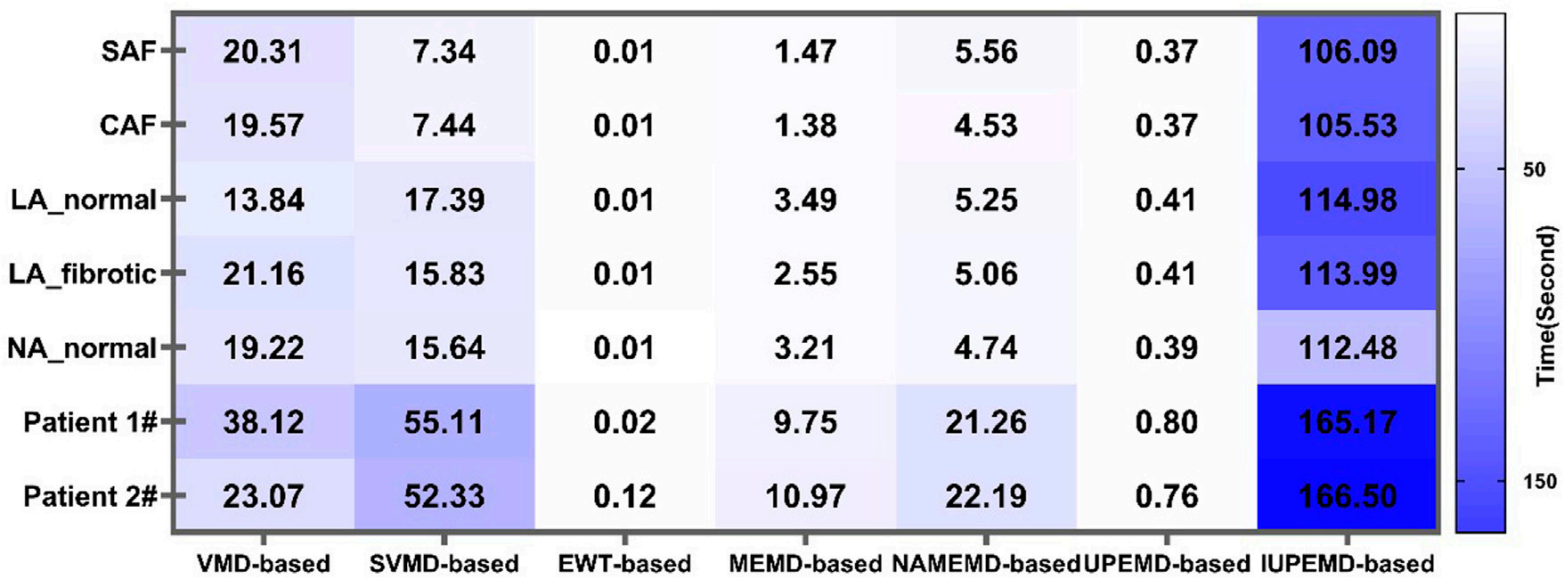
Decomposition time of ECGs by various EMD-based solutions for diverse datasets.

We counted the time taken by various EMD-based approaches for the decomposition of ECGs to assess the operational efficacy of various EMD-based solutions. The effect of datasets and methods on decomposition time is summarized in Figure 6.

On all datasets, EWT-based algorithms deconstruct the signal in the shortest time (no more than 0.12s), followed by UPEMD-based solutions (less than 0.81s), showing that these two schemes are more efficient than others. Due to the adaptive decomposition layers of the SVMD approach in different data sets, the SVMD-based method is not consistently more efficient than the VMD-based method in different simulated data sets. If the number of decomposition layers is greater than that of the VMD method, the length of time required will be greater. In contrast, if the number of adaptive decomposition layers is less than the predetermined value of the VMD method, the VMD method will consume less time. Due to the addition of a particular amount of white noise to the original signal in the NAMEMD-based solution (Rehman and Mandic, 2011), the time-frequency components of the signal become more diverse. Hence, more decomposed layers are required to get the final monotonic residual signal (Table 1). In comparison to the UPEMD-based solution, the IUPEMD-based solution must iteratively decompose the ECGs under various parameters (amplitude and phase number) using UPEMD and then determine the current optimal amplitude and the number of phases based on the principle of minimum orthogonality. Consequently, this method requires more time.

## 4 Discussion

This study employed five A F simulation datasets and two real AF patient datasets to evaluate the effect of various EMD-based solutions in ECGI. We obtain the Dis between the calculated and actual maximum DF, and maximum DP sites. What’s more, the CC and RDMS of the estimated DF and DP maps for different inverse operation methods are investigated for simulated AF datasets as well.

UPEMD-based IUPEMD-based and EWT-based solutions perform better than other EMD-based solutions on simulated datasets from the CC and RDMS of DF and DP maps, and their Dis between estimated and actual top 10 most probable driving sources is shorter.

In comparison to the IUPEMD-based solution, the UPEMD-based solution simply decompose ECGs with the assistance of a sinusoidal signal whose amplitude and phase are pre-determined (Wang et al., 2018), hence lowering the number of iterations and saving calculation time. The IUPEMD-based solution is inferior to the UPEMD-based solution, indicating that adding sinusoidal signals with the phases and amplitudes adaptively determined by the principle of minimum orthogonality to ECGs does not significantly improve the solution of the ECGI inverse problem. In real AF datasets, the maximum DF calculated by the UPEMD-based solution and the EWT-based solution are the closest to the actual maximum DF, indicating that the detected drivers are more precise using UPEMD-based and EWT-based solutions.

VMD-based and SVMD-based solutions are less satisfactory when compared to UPEMD-based and EWT-based technology. It demonstrates that the hypothesis that decomposition of the ECGs is translated into a variational problem is not conducive to the ECGI inverse problem (Dragomiretskiy and Zosso, 2014; Wei et al., 2021). In these two decomposition methods, the screened IMFs cannot distinguish the valuable components for the ECGI inverse problem well. Although the SVMD-based solution, as an improved version of the VMD-based solution, adds constraints when solving variational problems (Liu et al., 2022), it is not better than the VMD-based solution, in fact, it is marginally worse. Dependent on the different ECGs, the execution efficiency of VMD-based and SVMD-based solutions differs.

Similarly, neither the NAMEMD-based solution nor the MEMD-based solution show any advantages, indicating that the method of first mapping ECGs to a high-dimensional space and then performing multi-scale decomposition of their projected signals may not be appropriate for multi-scale solutions of ECGI. The Dis calculated by the NAMEMD-based solution is greater than that of the MEMD-based solution in the SAF, LA_fibrotic, and genuine AF datasets, demonstrating that NAMEMD-based, an enhanced MEMD-based solution, does not solve ECGI inverse problems more effectively. The execution efficiency of the NAMEMD-based solution is lower than that of the MEMD-based solution.

## 5 Limitations

In this paper, only a small amount of data was used to test these EMD-based approaches, and when these open data sets were collected, they may have had mistakes because of the limits of the measurement environment or the simulation environment.

We did not evaluate the effect of the geometric errors of the atrium and torso models on the performance of various EMD-based solutions. When AF occurs, not only does the electrical activity of the atrium change, but so does the atrial structure to a certain extent. The reconstructed portion of the structure, such as fibrosis, must be distinguished from the normal portion during the construction of the atrial geometric model and mesh division; moreover, the static atrial model is utilized in this study. Using a dynamic atrial model that incorporates the natural systolic and diastolic activities of the atrium may increase the accuracy of inverse calculations.

How the number of electrodes and electrode placements affect the outcomes of various EMD-based inverse methods has not been properly investigated. The two real AF datasets used have a limited number of surface electrodes. Although some researchers disagree that more surface electrodes make it more conducive to study the inverse problem (Bear et al., 2018; Gharbalchi No et al., 2020; Parreira et al., 2020), the degree of inaccuracy brought on by 54 electrodes in the study of the ECGI inverse problem deserves further in-depth investigation.

In conclusion, this study disregards the influence of the defects caused by the aforementioned challenges on the inverse solutions and focuses solely on the application of various EMD-based solutions to constrained datasets. To some extent, the findings are useful; however, a large number of clinical data have not confirmed them.

## 6 Conclusion

In conclusion, we study the application of different EMD-based solutions in ECGI, and the results have strong regularity. Considering the efficiency of algorithm execution, UPEMD-based and EWT-based solutions have prominent advantages and are easier to meet the needs of ECGI in clinical applications. The UPEMD-based solution, IUPEMD-based solution, and EWT-based solution are superior to others for distinct datasets. However, the IUPEMD-based solution’s running time is significantly longer than that of the UPEMD-based solution and EWT-based solution, causing the latter two more appropriate for clinical applications. This paper can serve as a reference for future researchers addressing relevant ECGI inverse problems by examining the use of various EMD-based solutions in ECGI.

## Data Availability

The original contributions presented in the study are included in the article/Supplementary Material, further inquiries can be directed to the corresponding author/s.

## Footnotes

1 This simulation dataset can be found in the [EDGAR] https://edgar.sci.utah.edu/human-cardiac-mapping.html.
