# Supplementary material for: Solving the inverse problem in electrocardiography imaging for atrial fibrillation using various time-frequency decomposition techniques based on empirical mode decomposition: A comparative study
Source: Front Physiol. 2022 Nov 2;13:999900. doi: 10.3389/fphys.2022.999900 (PMC9666773; doi:10.3389/fphys.2022.999900)
Supplement: Supplementary file 1 [file DataSheet2.DOCX]

# **Appendix**

## 2.2 EMD-based signal decomposition

### 2.2.1 Variational mode decomposition

Variational mode decomposition (VMD) defines IMF as a limited bandwidth amplitude modulation-frequency modulation (AM-FM) signal, requiring that the sum of each IMF's estimated bandwidths is the smallest, and that the sum of all IMFs is equal to the original signal,

$\min_{\{u_{k}\},\{w_{k}\}}\{\sum_{k} \left\| \partial_{t}[(\delta(t)+\frac{j}{\pi t})*u_{k}(t)]e^{-jw_{k}t} \right\|_{2}^{2}\}$ $s.t.\text{ }\sum_{k} u_{k}(t)=x(t)$ (1)

$\{u_{k}\}$、$\{w_{k}\}$ represent the *k-*th modal component and the corresponding center frequency, respectively($k=1,2,\cdots,K$)，$x(t)$ is the original signal. VMD introduces quadratic penalty factor $\alpha$ and Lagrangian multiplication operator $\lambda(t)$ to transform the above constrained variational problem into an unconstrained problem to find the optimal solution of the above formula.

$L\left( \left\{ u_{k} \right\},\left\{ w_{k} \right\},\lambda\right):=\alpha\sum_{k} \left\| \partial_{t}[(\delta(t)+\frac{j}{\pi t})*u_{k}(t)]e^{-jw_{k}t} \right\|_{2}^{2}+\left\| x(t)-\sum_{k} u_{k}(t) \right\|_{2}^{2}+\left\langle\lambda(t),x(t)-\sum_{k} u_{k}(t) \right\rangle$ (2)

The algorithm steps for VMD are as follows：

Step1: Set the initial iteration value for each modal component $\{u_{k}^{1}\}$, the center frequency $\{w_{k}^{1}\}$, and the Lagrange operator $\lambda^{1}$.

Step 2: Update the number of iterations $n\leftarrow n+1$.

Step 3: Update decomposition layers $k\leftarrow k+1$，update the *k-*th modal component $u_{k}$ and the center frequency $w_{k}$ according to formula (3) and (4) respectively.

$u_{k}^{n+1}\leftarrow\underset{\hat{u}_{k}}{argmin}L(\{u_{i<k}^{n+1}\},\{u_{i\geq k}^{n}\},\{\omega_{i}^{n}\},\lambda^{n})$ (3)

$\omega_{k}^{n+1}\leftarrow\underset{\hat{\omega}_{k}}{argmin}L(\{u_{i}^{n+1}\},\{u_{<k}^{n+1}\},\{\omega_{i\geq k}^{n}\},\lambda^{n})$ (4)

Step 4: If all of the decomposition layers have been updated, $k=K$, proceed to Step 5; otherwise, return to Step 3.

Step 5: Update the Lagrangian multiplication operator λ according to formula (5).

$\lambda^{n+1}\leftarrow\lambda^{n}+\tau(x-u_{k}^{n+1})$ (5)

Step 6: If equation (6)'s precision criterion is met, the iteration stop; otherwise, Step 2 is followed.

$\sum_{k} \frac{\left\| u_{k}^{n+1}-u_{k}^{n} \right\|_{2}^{2}}{\left\| u_{k}^{n} \right\|_{2}^{2}}<\varepsilon$ (6)

### 2.2.2 Successive variational mode decomposition

To make sure that the required modal components have no or less spectral overlap with other modal components and residual signals than in VMD, successive variational mode decomposition (SVMD) introduces a new penalty function. In other words, the energy of the residual signal is smallest at the center frequency of the modal component $u_{k}(t)$, and the energy of other frequencies is smallest there as well. Both the original and modified punishment functions are represented as

$J_{1}=\sum_{k} \left\| \partial_{t}[(\delta(t)+\frac{j}{\pi t})*u_{k}(t)]e^{-jw_{k}t} \right\|_{2}^{2}$ (7)

$J_{2}=\sum_{i}^{K-1} \left\| \beta_{i}(t)*u_{k}(t) \right\|_{2}^{2}$ (8)

$J_{3}=\left\| \beta_{k}(t)*r_{K}(t) \right\|_{2}^{2}$ (9)

Where $\beta_{k}(t)$ represents the impulse response of the filter with a frequency response $w_{k}$. The final solution objective of SVMD is expressed as formula (9), where $\alpha$ is the penalty factor that balances *J_1_*, *J_2_* and *J_3_*, which can be solved by Lagrangian method.

$\min_{u_{k},w_{k},r_{K}}=\{\alpha J_{1}+J_{2}+J_{3}\}$ (10)

The decomposition process of SVMD is as follows:

Step 1: Initialize the penalty factor $\alpha$, the iteration termination accuracy $\varepsilon_{1}$ and $\varepsilon_{2}$, the additive white noise $\sigma^{2}$, and the decomposition layers $k=0$.

Step 2: $k\leftarrow k+1$，set the initial iteration value of the modal component $\hat{u}_{k}^{1}$, the center frequency $\omega_{k}^{1}$ and Lagrangian operator $\hat{\lambda}^{1}$，set iteration number n=0；

Step 3: Update iteration number $n\leftarrow n+1$，and update modal components $\hat{u}_{k}^{n}$、the center frequency $\hat{w}_{k}^{n}$ and the Lagrange operator $\hat{\lambda}^{n}$ based on formulas (11), (12) and (13);

$\hat{u}_{k}^{n+1}(\omega)=\frac{\hat{x}(\omega)+\alpha^{2}(\omega-\omega_{k}^{n})^{4}\hat{u}_{k}^{n}(\omega)+\frac{\hat{\lambda}(\omega)}{2}}{[1+\alpha^{2}(\omega-\omega_{k}^{n})^{4}][1+2\alpha(\omega-\omega_{k}^{n})^{2}+\sum_{i}^{k-1} \frac{1}{\alpha^{2}(\omega-\omega_{i})^{4}}]}$ (11)

$\omega_{k}^{n+1}=\frac{\int_{0}^{\infty} \omega\left| \hat{u}_{k}^{n+1}(\omega) \right|^{2}d\omega}{\int_{0}^{\infty} \left| \hat{u}_{k}^{n+1}(\omega) \right|^{2}d\omega}$ (12)

$\hat{\lambda}^{n+1}=\hat{\lambda}^{n}+\tau[\hat{x}(\omega)-(\hat{u}_{k}^{n+1}(\omega)+[\frac{\alpha^{2}(\omega-\omega_{k}^{n+1})^{4}(\hat{x}(\omega)-\hat{u}_{k}^{n+1}(\omega)-\sum_{i}^{k-1} \hat{u}_{i}(\omega)+\frac{\hat{\lambda}(\omega)}{2})-\sum_{i}^{k-1} \hat{u}_{i}(\omega)}{1+\alpha^{2}(\omega-\omega_{k}^{n+1})^{4}}]+\sum_{i}^{k-1} \hat{u}_{i}^{n+1}(\omega))]$ (13)

Step 4: Check to see if the formula (14) is satisfied; if so, move on to Step 5; if not, return to Step 3.

$\frac{\left\| \hat{u}_{k}^{n+1}-\hat{u}_{k}^{n} \right\|_{2}^{2}}{\left\| \hat{u}_{k}^{n} \right\|_{2}^{2}}<\varepsilon_{1}$ (14)

Step 5: If the precision condition of formula (15) is satisfied, the decomposition is terminated; otherwise, return to Step 2.

$\frac{\left| \sigma^{2}-\frac{1}{T}\left\| x(t)-\sum_{k} u_{k}(t) \right\|_{2}^{2} \right|}{\sigma^{2}}<\varepsilon_{2}$ (15)

### 2.2.3 Multivariate empirical mode decomposition

The decomposition flowchart of multidimensional empirical mode decomposition (MEMD) is as follows:


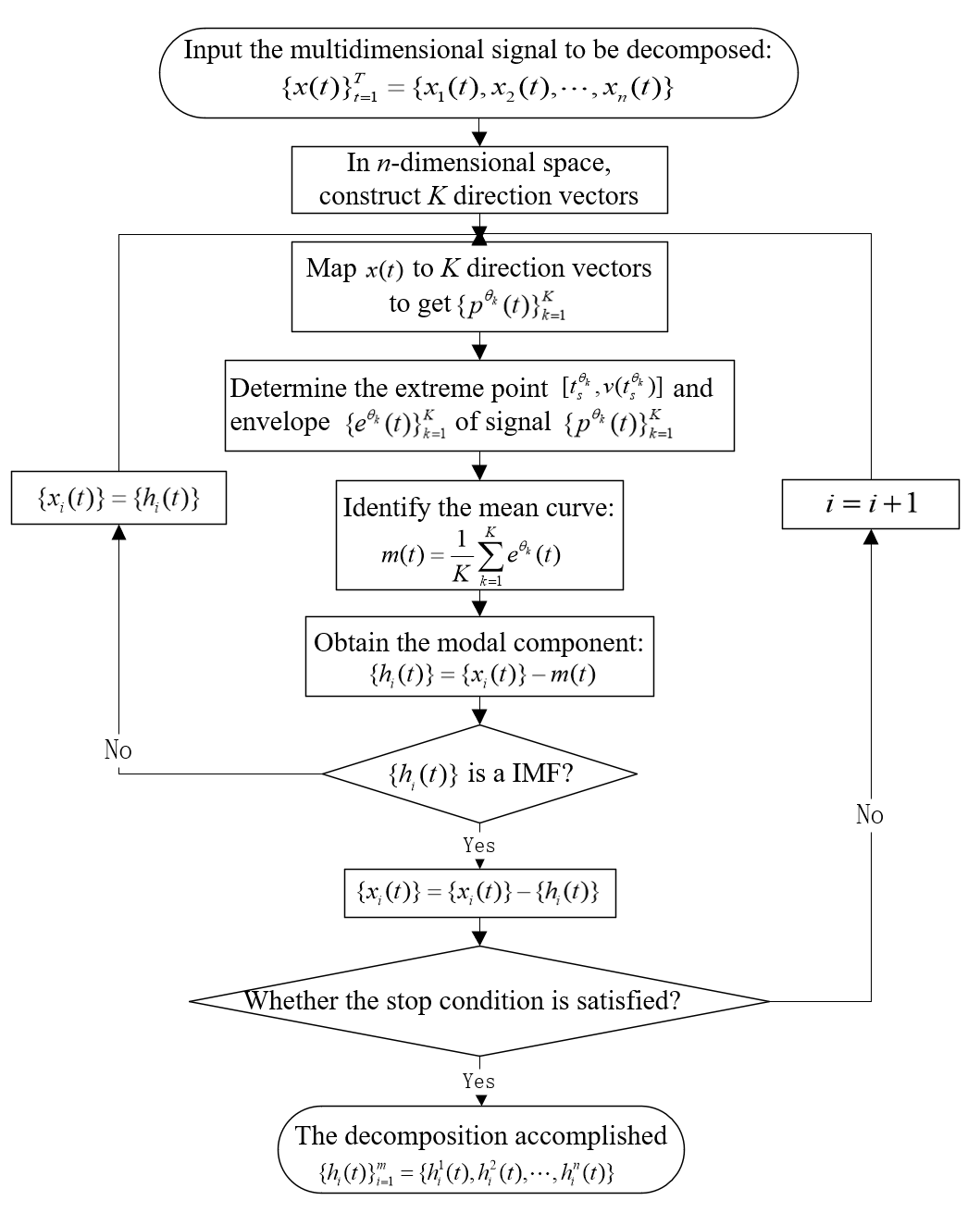


Figure 1. The decomposition flowchart of MEMD

### 2.2.4 Noise-assisted multivariate empirical mode decomposition

Noise-assisted MEMD (NAMEMD) is the process of combining the white noise $\{v(t){\}}_{t=1}^{T}$ of *M* channels with the input signal $\{x(t){\}}_{t=1}^{T}$ before performing MEMD.

The following are the NAMEMD execution steps：

Step1: Splicing *M* channels of white noise $\{v(t){\}}_{t=1}^{T}$ into the input signal $\{x(t){\}}_{t=1}^{T}$ to create a new input signal$\{Z(t){\}}_{t=1}^{T}=\{x_{1}(t),x_{2}(t),\cdots,x_{n}(t),v_{1}(t),v_{2}(t),\cdots,v_{m}(t)\}$；

Step2：Decompose $\{Z(t){\}}_{t=1}^{T}$ by MEMD；

Step3: Get the IMF of the *n*-channel input signals after discarding the IMF corresponding to the *M* channels' white noise.

### 2.2.5 Uniform phase empirical mode decomposition


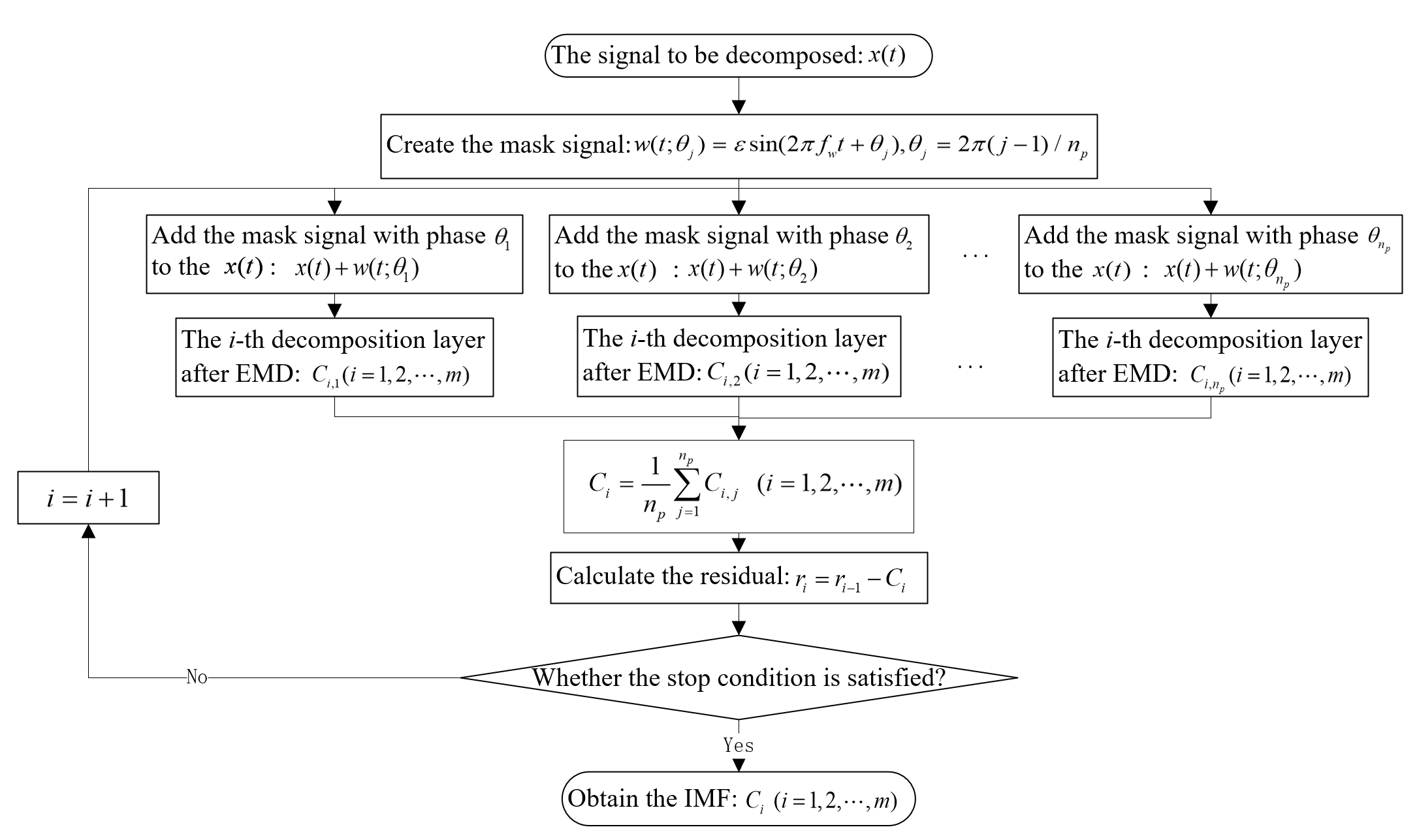


Figure 2. The flowchart of UPEMD

Where the relationship between the phase type $\theta_{j}$ and the phase number $n_{p}$ can be described as$\theta_{j}=2\pi(j-1)/n_{p}(j=1,2,\cdots,n_{p})$.

### 2.2.6 Improved uniform phase empirical mode decomposition


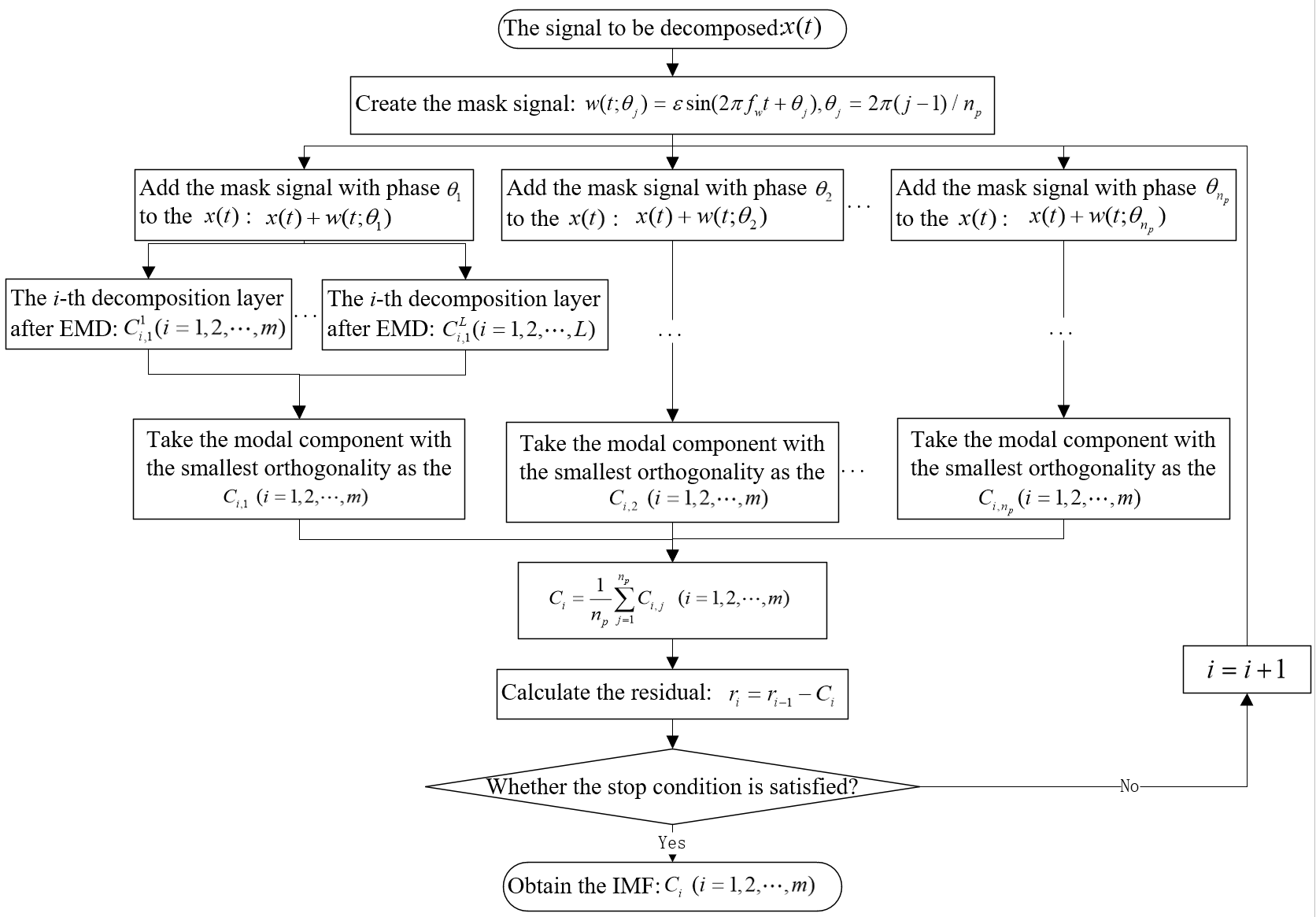


Figure 3. The flowchart of IUPEMD

### 2.2.7 Empirical wavelet transform

The following are the Empirical wavelet transform (EWT) implementation steps:

Step1：Apply the Fourier transform to the raw signal to find maxima in the spectral range $[0,\pi]$ and determined the segmentation boundaries. Denote the angular frequency corresponding to the first *N* maximum values in the frequency domain as $\Omega_{n}(n=1,2,\cdots,N)$，and the spectrum division boundary as $\omega_{n}=(\Omega_{n+1}+\Omega_{n})/2$;

Step2：Divide the Fourier spectrum in the range of $[0,\pi]$ into *N* continuous intervals with reference to the division boundary in Step1. Each interval can be expressed as $I_{n}=\left[ \omega_{n-1},\omega_{n} \right]$, where $n=1,2,\cdots,N$.

Step3: A bandpass filter bank $I_{n}$ is constructed on each interval. When $n>0$, the empirical wavelet function $\psi_{n}(\omega)$ and the empirical scale function $\varphi_{n}(\omega)$ are shown in formulas (16) and (17), respectively.

$\psi_{n}(\omega)=\left\{\begin{matrix} 1,\text{ }\left|\omega\right|\leq\omega_{n}-\tau_{n}\text{ } \\ \cos\{\frac{\pi}{2}\beta[\frac{1}{2\tau_{n+1}}(\left|\omega\right|-\omega_{n+1}+\tau_{n+1})]\},\text{ }\omega_{n+1}-\tau_{n+1}\leq\left|\omega\right|\leq\omega_{n+1}+\tau_{n+1} \\ \cos\{\frac{\pi}{2}\beta[\frac{1}{2\tau_{n}}(\left|\omega\right|-\omega_{n}+\tau_{n})]\},\text{ }\omega_{n}-\tau_{n}\leq\left|\omega\right|\leq\omega_{n}+\tau_{n}\text{ } \\ 0,\text{ }others \end{matrix}\right.$ (16)

$\varphi_{n}(\omega)=\left\{ \begin{aligned} 1,\text{ }\left| \omega\right|\leq\omega_{n}-\tau_{n}\text{ } \\ \cos\{\frac{\pi}{2}\beta[\frac{1}{2\tau_{n}}(\left| \omega\right|-\omega_{n}+\tau_{n})]\},\text{ }\omega_{n}-\tau_{n}\leq\left| \omega\right|\leq\omega_{n}+\tau_{n} \\ 0,\text{ }others \end{aligned} \right.$ (17)

Step 4：As indicated in Equation (18), each single-component parts $x_{k}(t) (k=1,2,3\cdots)$ with frequencies ranging from low to high are obtained using wavelet decomposition. The wavelet transform's detail coefficient and approximation coefficient, respectively, can be stated as Equation (19) and (20).

$\left\{ \begin{aligned} x_{0}(t)=W(0,t)*\varphi_{1}(t) \\ x_{k}(t)=W(k,t)*\psi_{k}(t) \end{aligned} \right.$ (18)

$W(k,t)=\left\langle x,\psi_{k} \right\rangle=\int x(\tau)\overline{\psi_{k}(\tau-t)}d\tau$ (19)

$W(0,t)=\left\langle x,\varphi_{1} \right\rangle=\int x(\tau)\overline{\varphi_{1}(\tau-t)}d\tau$ (20)

Step 5：Reconstruct the signal decomposed in Step 4 using the formula (21)：

$x(t)=W(0,t)*\varphi_{1}(t)+\sum_{k=1}^{N} W(k,t)*\psi_{k}(t)$ (21)
