# Supplementary material for: Solving the inverse problem in electrocardiography imaging for atrial fibrillation using various time-frequency decomposition techniques based on empirical mode decomposition: A comparative study
Source: Front Physiol. 2022 Nov 2;13:999900. doi: 10.3389/fphys.2022.999900 (PMC9666773; doi:10.3389/fphys.2022.999900)
Supplement: Supplementary file 2 [file DataSheet1.DOCX]

**Figures**

Figure 1. EMD-based solution to the inverse problem in ECGI


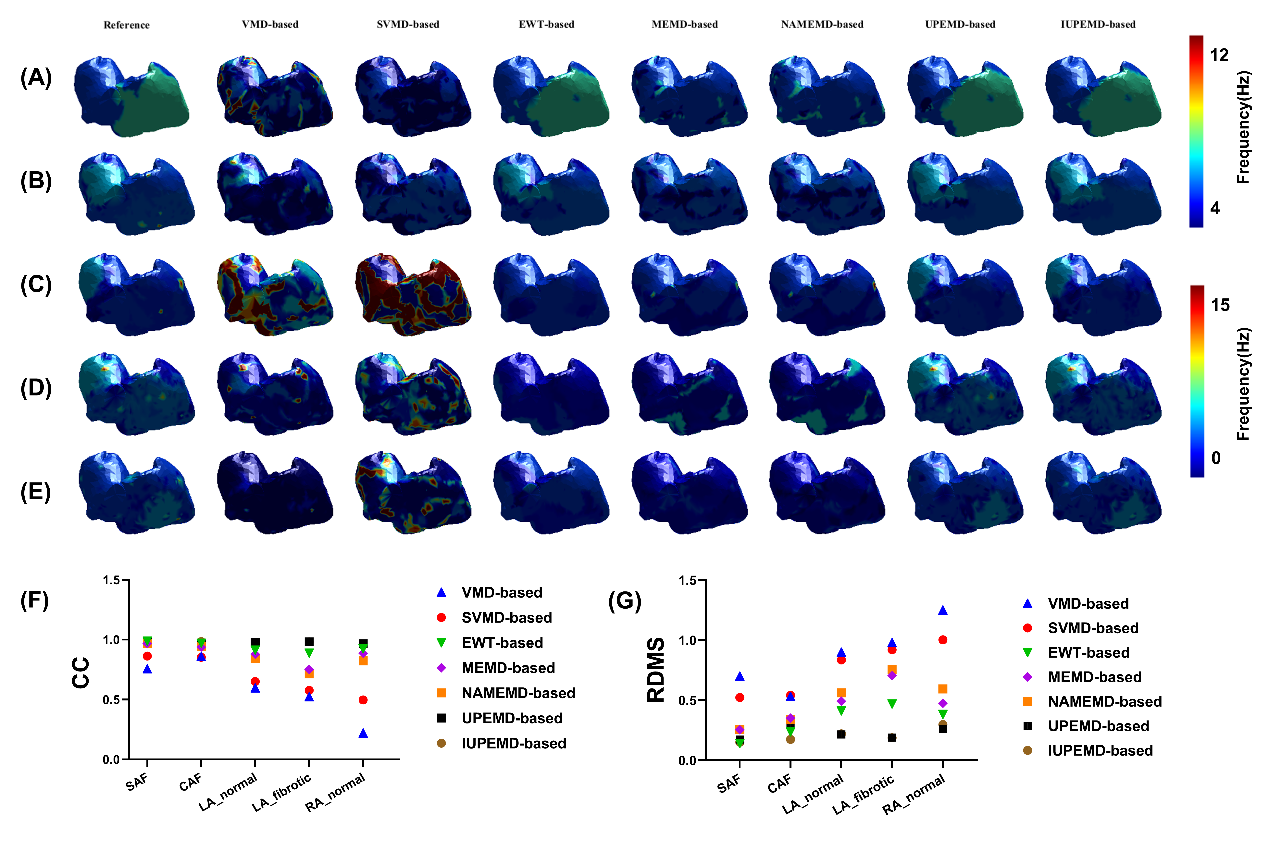


Figure 2. The DF maps using various EMD-based methods under the datasets SAF, CAF, LA_normal, LA_fibrotic, and RA_normal are represented by (A)~(E), respectively. For each separate dataset, the reference DF map is in the first column, followed by the DF maps for the VMD-based, SVMD-based, EWT-based, MEMD-based, NAMEMD-based, UPEMD-based, and IUPEMD-based solutions, starting with the second column. On each model, a distinct color denotes the DF at that place. The RDMS (G) and CC (F) between the computed EMD-based algorithm and the real DF map for each EMD-based solution are shown as well.


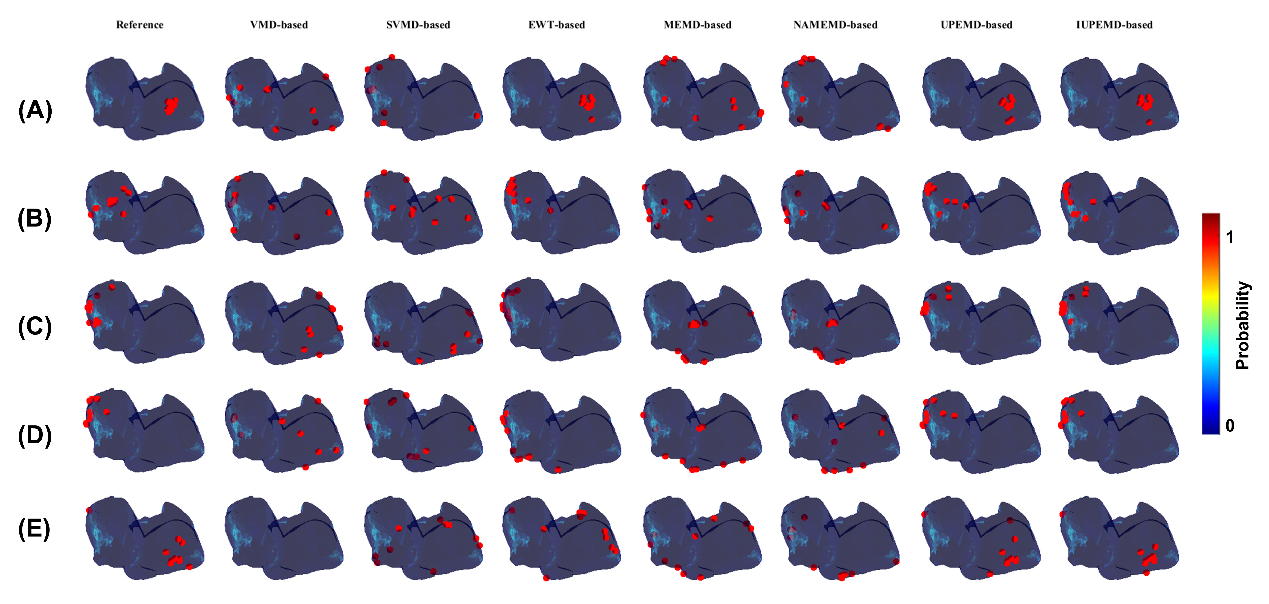


Figure 3. DP maps (red circles mark the 10 locations with the highest probability of driver location). Among them, (A)~(E) represent the DP maps generated by various EMD-based algorithms for SAF, CAF, LA_normal, LA_fibrotic, and RA_normal, which from left to right correspond to the DP maps for VMD-based, SVMD-based, EWT-based, MEMD-based, NAMEMD-based, UPEMD-based, and IUPEMD-based solutions.


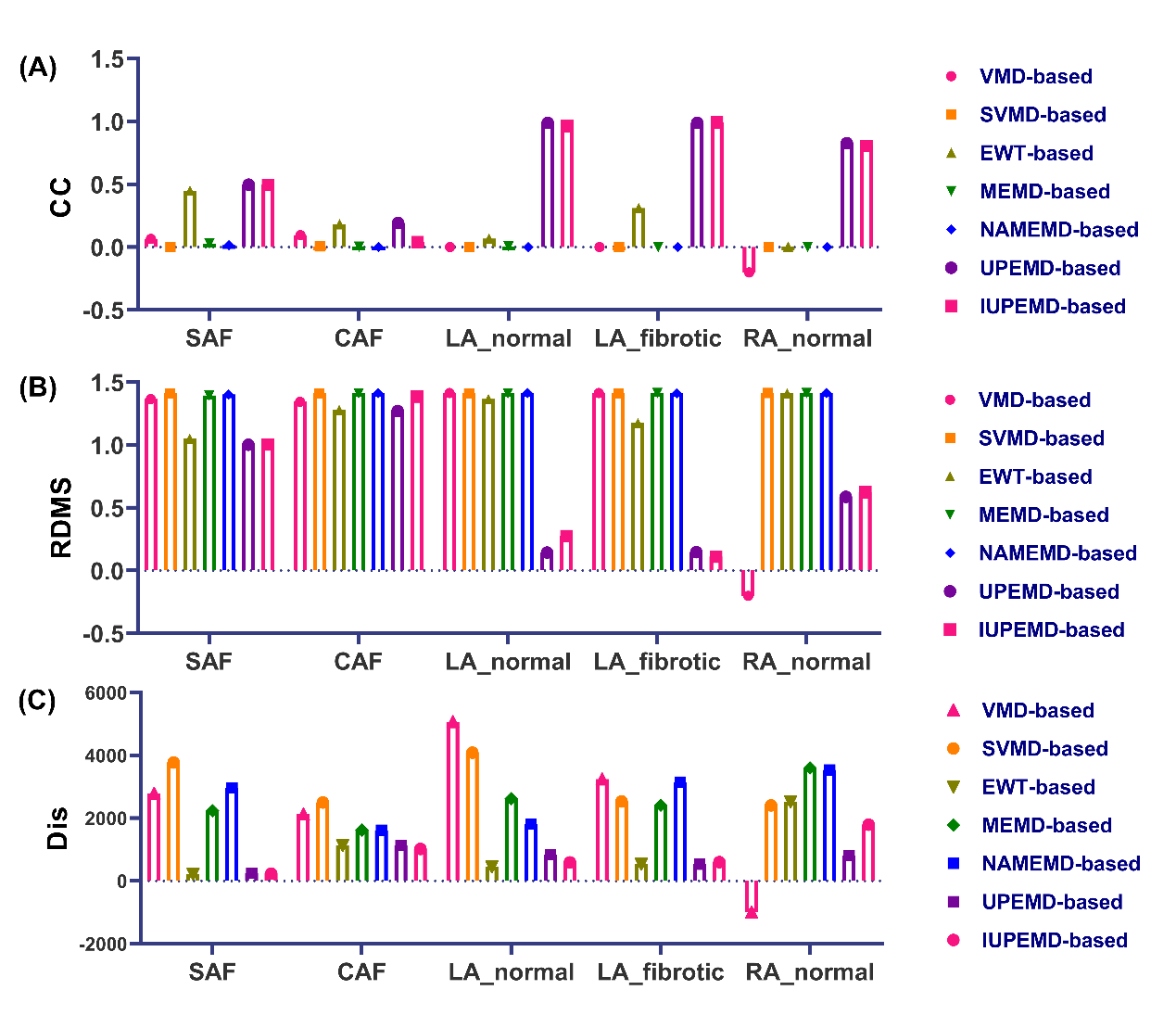


Figure 4. Quantitative evaluation of the DP map: (A) CC (B) RDMS (C) Dis (top 10 greatest possible drivers). The horizontal axis displays, from left to right, the quantitative indications of VMD-based, SVMD-based, EWT-based, MEMD-based, NAMEMD-based, UPEMD-based, and IUPEMD-based solutions for every dataset.


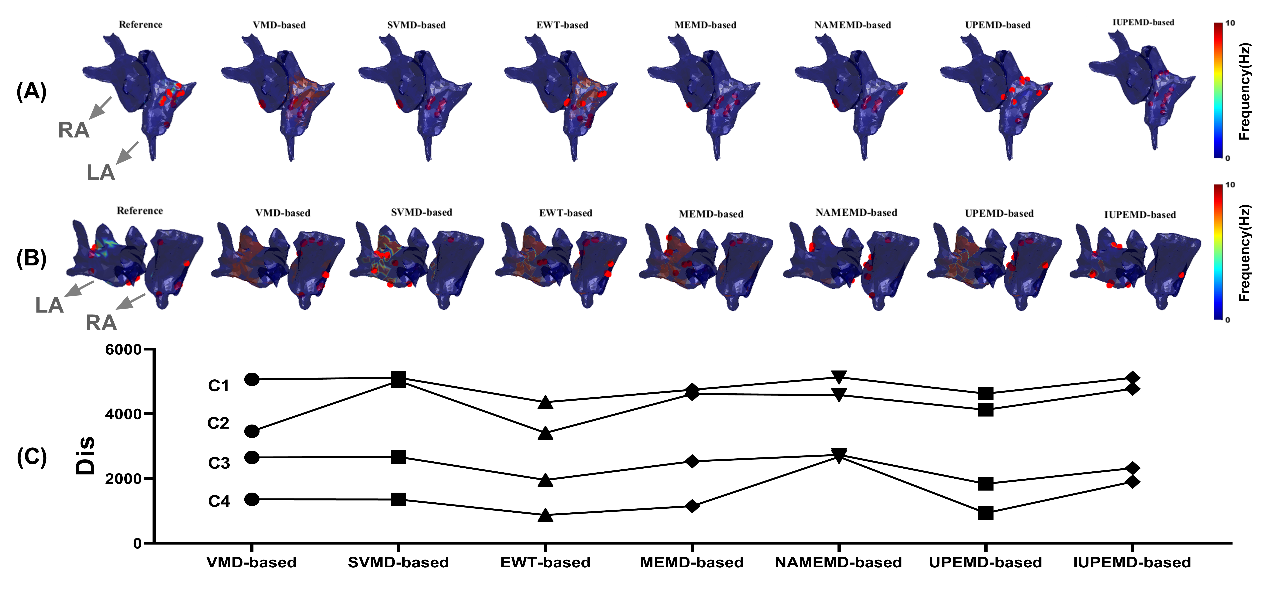


Figure 5. DF maps of two real patients with AF (red dots mark the 10 locations with the highest DF; both patients' LA and RA are indicated by grey arrows). (A) DF maps for Patient 1#; (B) DF maps for Patient 2#; (C) The Dis between the computed and actual DF maps: C1, for Patient 1#, the Dis between the estimated and actual 62 maximum DF positions for various EMD-based solutions; C2, for Patient 2#, the Dis between the estimated and actual 73 maximum DF positions; C3, for Patient 1#, the Dis between the first 10 real and estimated maximum DF positions; C4, for Patient 2#, the Dis between the first 10 real and inversely calculated maximum DF positions.


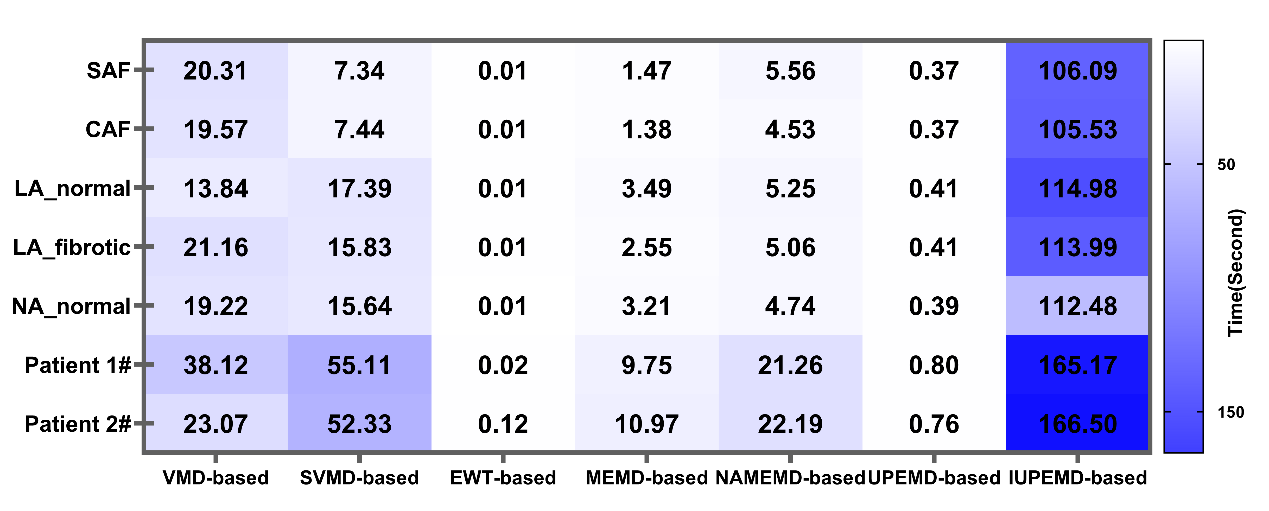


Figure 6. Decomposition time of ECGs by various EMD-based solutions for diverse datasets
